# Study on Mechanical Properties of Concrete Using Basalt-Based Recycled Aggregate and Varying Curing Conditions

**DOI:** 10.3390/ma15134563

**Published:** 2022-06-29

**Authors:** Hong-Beom Choi, Jin-O Park

**Affiliations:** Construction Test & Assessment Center, Construction Test and Certification Department, Korea Institute of Civil Engineering and Building Technology, Goyang-si 12345, Korea; hongbeomchoi@kict.re.kr

**Keywords:** basalt, recycled aggregate, curing, fly ash

## Abstract

To replace porous basalt, the mechanical properties of concrete with recycled resources and durability improvement were analyzed in this study. The analysis was based on the quality improvement of recycled aggregate, use of fly ash, and changes in curing conditions. Basalt aggregate (BA) with a 3% water absorption, raw recycled aggregate and basalt (RRA), and improved recycled aggregate and basalt (PRA) were the main experimental variables. As PRA was applied to concrete, the compressive strength was lower than that of the specimen comprising BA in the normal strength region, but the modulus of elasticity (22.9 GPa) was equivalent or higher. The initial drying shrinkage increased because of the use of basalt-based recycled aggregate (B-RA). The drying shrinkage of PRA was similar to that of BA with an average difference of <7% as the age increased. The specimens subjected to steam curing exhibited the lowest drying shrinkage. These results showed that pores in the old paste of recycled aggregate increased freeze–thaw resistance because of the increase in the spacing factor. Although the PRA did not satisfy the quality criteria, the efficient use of recycled aggregate achieved an equivalent or higher performance than that of concrete comprising BA and improved durability.

## 1. Introduction

Industrial development has resulted in the rapid rise of concrete-based buildings accompanied by environmental degradation due to the use of numerous natural resources, such as aggregate. The collection of natural resources led to the destruction of rivers and mountains, resulting in a lack of resources [1,2,3]. Old buildings are dismantled for reconstruction reasons, and global construction waste amounts to 1.3 billion tons/year [4,5]. Various studies have investigated the use of recycled aggregates as substitutes for natural resources and for treating construction waste to reduce the environmental burden resulting from massive waste generation [6,7,8,9,10]. The physical properties of concrete are not significantly affected if recycled aggregate replaces less than 30% of coarse aggregate during concrete production. However, the results differ depending on research conditions; various studies have investigated how recycled aggregate concrete is affected by the characteristics of the parent rock included in coarse recycled aggregate [11,12,13]. In addition, various quality improvement studies have been conducted using industrial by-products, such as blast furnace slag and fly ash (FA), as supplementary cementitious materials (SCMs) considering the strong alkalinity of the old cement paste of recycled aggregate [14,15]. Several studies have proposed the use of recycled aggregates as representative alternative aggregates, and attempts have been made to address the critical durability problem using fibers and improved curing methods [16,17].

Meanwhile, environmental destruction is strongly resisted in Jeju Island, Korea, which has been designated as a world natural heritage for its pristine nature. Thus, it is difficult to secure basic resources while plans are underway to construct large-scale infrastructure, such as new airports and ports. Environmental preservation and the limited industrial structure of the island also limit alternative aggregate sources, and construction waste is an effective resource in this situation. The recycled aggregate of Jeju Island, however, has yet to received quality certification for concrete in Korea, but it has been used as aggregate for road subbase with a low added value. This limited use is related to the quality of the natural basalt aggregate from the area, which is used as the parent rock of waste concrete in Jeju Island, although technology is lacking for removing cement paste and mortar. Jeju Island was formed from volcanic activity; thus, basalt is the single natural aggregate in the island [18,19].

According to the literature, basalt is classified into 16 types through petrological classification, and its quality varies depending on the environment and region [20]. Numerous studies have used overseas basalt as high-strength aggregate because of its low porosity. Basalt found in Jeju Island has a relatively high porosity, and the water absorption of basalt aggregate ranges widely from 0 to 10% [21,22,23,24,25,26,27]. Basalt from aggregate quarries has a water absorption of approximately 3%. Recycled aggregate containing basalt exhibits relatively high water absorption; it has higher water absorption than other rock aggregates distributed in the inland areas of Korea (approximately 1%) [28]. In addition, an increase in specific surface area through the internal and external basalt pores can significantly degrade the quality of recycled aggregate because the increase adversely affects the removal of old paste and old mortar from waste concrete.

In the exploration of basalt as coarse aggregate, numerous international studies have investigated the use of basalt in high-strength concrete as well as concrete characterization according to rock classification. However, domestic studies on porous basalt are insufficient, as well as studies on recycled aggregate concrete based on porous basalt [29,30,31,32]. A previous study found that concrete of 50 MPa or higher can be produced depending on the quality of basalt-based recycled aggregate (B-RA) while the performance degradation of such concrete is relatively low [33]. The application of the concrete was problematic because of the low aggregate collection rate due to the repeated peeling intended to enhance the recycled aggregate quality. Therefore, it is crucial to determine effective methods for enhancing the recycled aggregate collection rate and durability, which affect the utilization of recycled aggregate.

Although new studies with various novelty have been conducted, they have not been applied to research or practical applications. This study intends to apply the real goals and variables to industry. The aim of this study was to examine the industrial application of recycled aggregate for concrete in Jeju Island. We examined the applicability of recycled aggregate with improved collection rate by simplifying the recycled aggregate production process and varying the mechanical properties and durability under three curing conditions (water, dry air, and steam) for various applications. FA was used to minimize the quality degradation caused by simplifying the recycled aggregate production process, and experiments were performed to examine the quality change that induces long-term pozzolanic reaction. The characteristics of fresh and hardened concrete were analyzed as basic data for the industrial application of recycled aggregate in Jeju Island.

## 2. Experiments and Methods

### 2.1. Experimental Plan

The experimental overview of this study is presented in Table 1. The experimental variables include the aggregate type, FA substitution rate, and curing condition. For the three variables with three factors, nine mixtures were set using the Taguchi experimental design method, as detailed in Table 2. The aggregate used in the experiment was pre-wetted in water for 24 h, considering the high absorption rate of the aggregate, and then the experiment was conducted by correcting moisture. The measured experimental quantities were the slump, air content, compressive strength, modulus of elasticity, drying shrinkage, freezing and thawing, and pore distribution.

### 2.2. Materials

**Figure 1:**
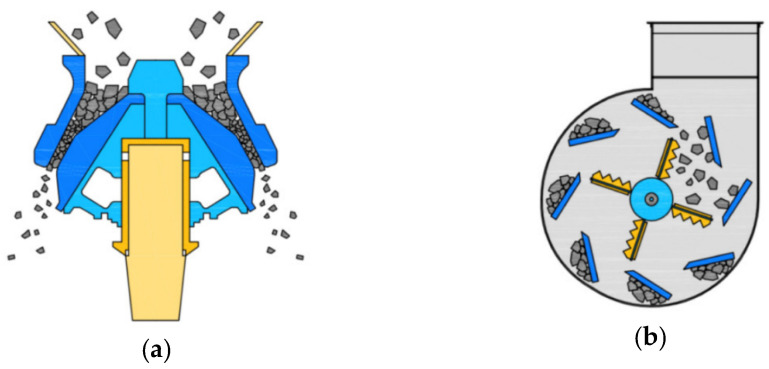
Manufacturing process of the recycled aggregate: (**a**) Type A; (**b**) Type B.

**Figure 2:**
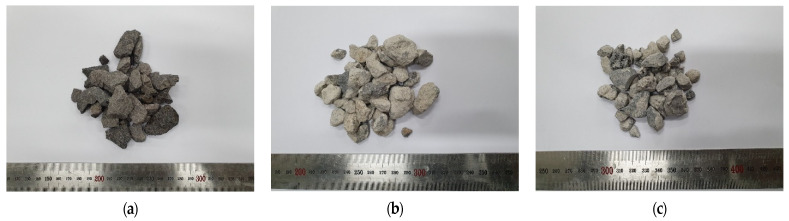
Coarse aggregates: (**a**) BA; (**b**) RRA; (**c**) PRA.

**Figure 3:**
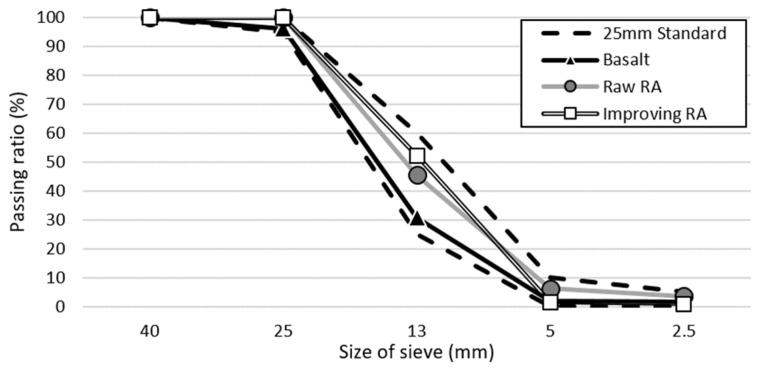
Grading of coarse aggregate.

In the experiment, we used Type I ordinary Portland cement from “S” company (Dangjin City, Korea) and FA from “Y” thermal power plant (Incheon City, Korea). The physical properties of the materials are listed in Table 3 and Table 4 while their chemical properties are listed in Table 5. The cement satisfied the Type I Portland cement standards of ASTM C150:2020 while the FA satisfied the Class F FA standards of ASTM C618:2019 [34,35]. Among the coarse aggregates, basalt aggregate (BA), which is the natural aggregate in Jeju Island, was obtained from Namwon-eup, Seogwipo City, and 5- to 25-mm aggregates satisfying the ASTM C33:2018 concrete aggregate were used [36]. Recycled aggregate was produced using Types A and B manufacturing processes, as illustrated in Figure 1. For Type A, the aggregates were crushed by the transmission of an impact force. For Type B, the aggregates were first placed in buckets in the top section of the equipment and then dropped on rotating blades. Here, the aggregates were produced by applying an impact force to the aggregate surface through high-speed rotating blades and peeling of the old paste. RRA was produced by Type A while PRA was produced by applying Type B as a secondary process to RRA. Depending on the characteristics of the process, Type A has a high aggregate collection rate, and Type B improves the properties of aggregate such as aggregate strength and water absorption. Figure 2 shows the coarse aggregates used, and their physical properties are presented in Table 6. Figure 3 shows the grading curve of each aggregate. RRA and PRA were mixed with BA for use. The chemical compositions are listed in Table 7.

### 2.3. Methods

**Figure 4:**
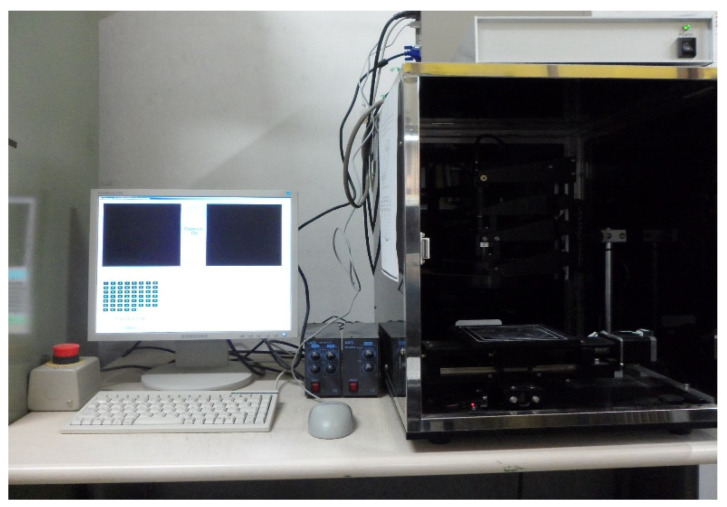
Image analysis device (HF-MA C01).

We conducted experiments in accordance with ASTM standards to analyze the fresh properties and hardened properties of the concrete. Accordingly, the slump of the fresh concrete was measured twice with a slump cone (base diameter, 200 mm; top diameter, 100 mm; height, 300 mm) according to the ASTM C143:2020 [37] specifications. The air content of fresh concrete was measured with a Type-B air meter with a capacity of 7.0 L according to the ASTM C231:2017 [38] specifications. The concrete was maintained in a sealed state after the initial measurement. The compressive strengths of the cylindrical concrete specimens (diameter, 100 mm; height, 200 mm) were then determined with a stress rate of 0.25 MPa/s for three or more specimens at ages of 3 d, 7 d, 28 d, and 1 year. The compressive strength was measured in accordance with ASTM C39:2020 [39]. The modulus of elasticity was measured with a loading rate of 0.25 MPa/s for three or more specimens by attaching strain gauges to the 28-day-old cylindrical specimens; the measurement was performed by applying load in accordance with ASTM C469:2014 [40] (concrete cylindrical specimen; static modulus of elasticity) and by using the Poisson’s ratio test method. The drying shrinkage of three specimens was measured by attaching strain gauges PL-60-11 (Tokyo Sokki Kenkyujo Co., Ltd., Tokyo, Japan) to the surface of the 100 × 100 × 400-mm cuboid specimens after demolding at 7 d of curing, in accordance with the concrete drying shrinkage test method of ASTM C596:2018 [41]. In both strain-gauge applications, a data logger (Tokyo Sokki Kenkyujo Co., Ltd., Tokyo, Japan) was used to acquire data under constant temperature and humidity conditions of 20 ± 2 °C and 60 ± 5%, respectively. The freeze–thaw test was conducted using 100 × 100 × 400-mm specimens in accordance with the ASTM C666:2017 Standard Test Method for Resistance of Concrete to Rapid Freezing and Thawing. The temperature change from 4 ± 2 °C to −18 ± 2 °C was set as one cycle, and the resonance frequency was measured every 30 cycles until 200 cycles [42]. The pore structure of hardened concrete was analyzed with the linear movement method and improved pore number measurement method specified in the ASTM C457 Standard Test Method for Microscopical Determination of Parameter of the Air-Void System in Hardened Concrete. The pore structure was analyzed through the microscope of the image analysis equipment (HF-MA C01) shown in Figure 4 [43].

## 3. Experimental Results

### 3.1. Slump

**Figure 5:**
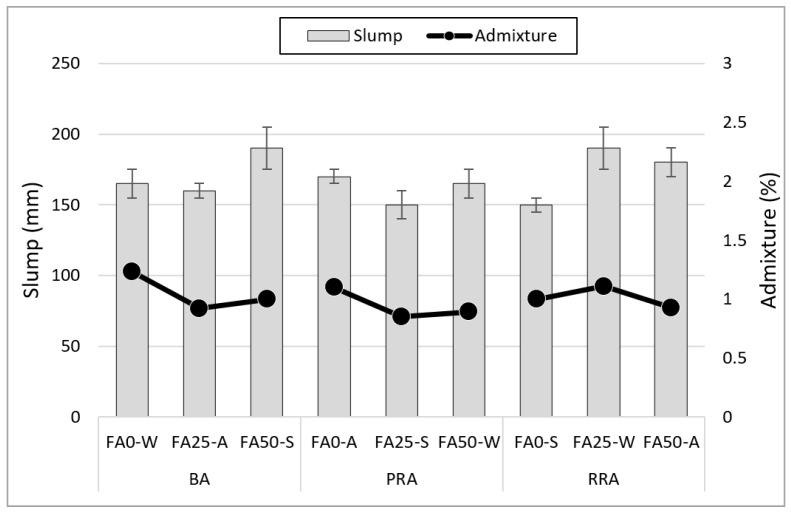
Slump of fresh concrete.

Figure 5 illustrates the concrete slump according to the coarse aggregate type and FA substitution rate. All the specimens satisfied the target slump condition of 175 ± 25 mm, and the superplasticizer amount did not differ significantly as the difference was approximately 1%. Compared to the superplasticizer amount used, the mixtures comprising BA exhibited a relatively low slump, indicating that the low sphericity of BA affected the slump. When FA was used, an additional amount of the superplasticizer was required compared to the reference mixture at a substitution rate of 25%; a higher slump than that of the reference mix was observed at a substitution rate of 50%. Previous studies have shown that FA tends to decrease slump when FA replaces weight because FA density is lower than that of cement, and the specific surface area of the binder increases [44,45]. When a substitution rate of 50% was used, the ball bearing effect of using FA had a greater impact.

### 3.2. Air Content

**Figure 6:**
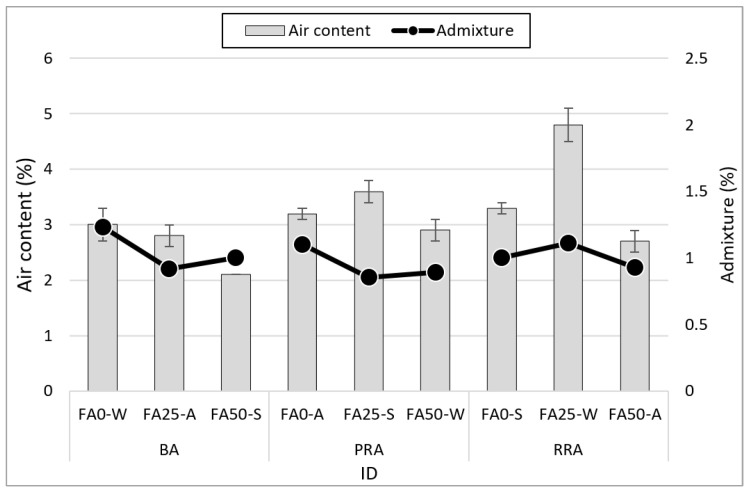
Air content of fresh concrete.

Figure 6 illustrates the air content measurement results for fresh concrete according to the coarse aggregate type and FA substitution rate. The recycled aggregate mixtures exhibited higher air contents than those of the BA mixtures, and the air content tended to decrease as the FA substitution rate increased. The tendency was not significant at an FA substitution rate of 25%, and the PRA FA25 mix exhibited the highest air content, which was due to the influence of the superplasticizer content. Regardless of the aggregate type, air content decreased at an FA substitution rate of 50%. This phenomenon can be attributed to the air adsorption occurring as a large amount of FA was used (the unburned amount increased). Regardless of the use of RRA and PRA, which had 5.4 and 2.0% higher water absorption than that of BA, air content did not increase significantly despite the application of pressure because of aggregate pre-wetting before mixing.

### 3.3. Compressive Strength

**Figure 7:**
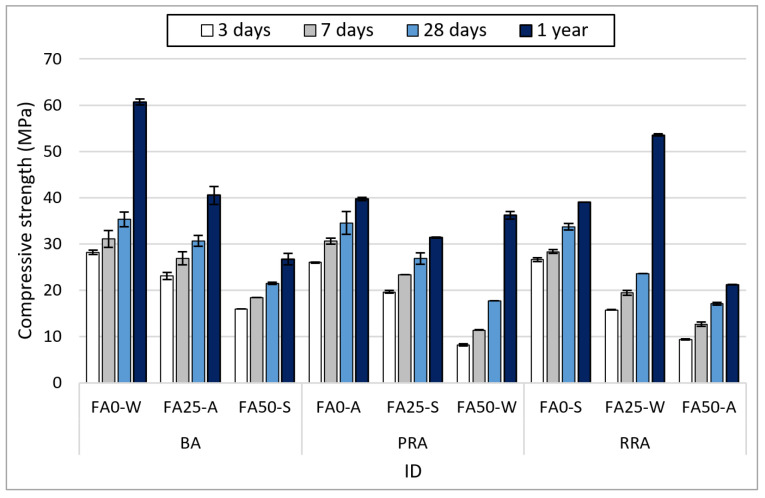
Compressive strength of concrete.

Figure 7 illustrates the compressive strength measurement results at 3 d, 7 d, 28 d, and 1 year of age for concrete containing recycled aggregate based on Jeju basalt. Different tendencies were observed depending on the aggregate type, FA substitution rate, and curing condition. First, an increase in FA substitution rate caused a reduction in compressive strength until 28 d. At 1 year, however, specimens under water curing exhibited a high strength. Particularly, PRA FA25-W, which used water curing and an FA substitution rate of 25%, exhibited a high strength of 55.1 MPa; RRA FA50-W, which used an FA substitution rate of 50%, exhibited 38 MPa. This phenomenon occurred because the strength was slowly developed after 28 d as large amounts of FA (25 and 50%) were used in the concrete. For RRA FA50-W, however, the low aggregate strength of RRA also had an impact, as well as the lack of alkalinity that develops strength with 50% FA.

**Figure 8:**
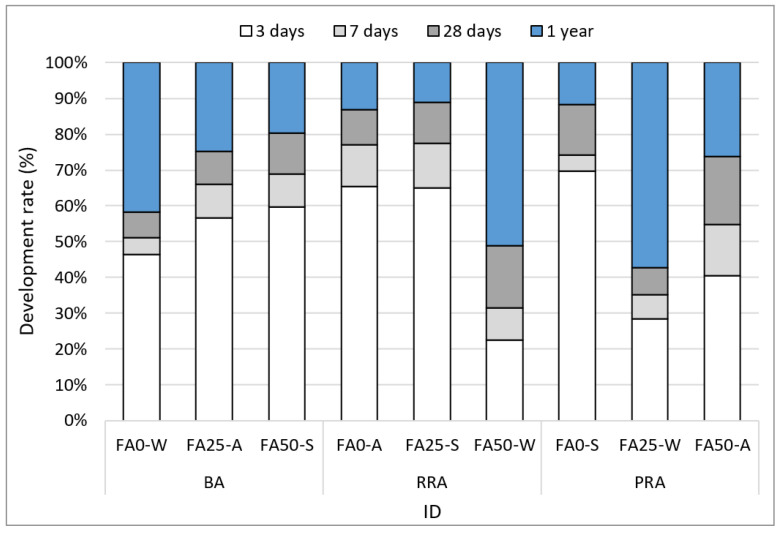
Development rate on compressive strength of concrete.

The compressive strength tendency in relation to curing can be more clearly observed from the compressive strength development rate based on a one-year strength, as illustrated in Figure 8. The lowest and highest strength development rates were exhibited by water curing (W) and steam curing (S), respectively. In addition, the strength development rate increased as the FA content increased, and the strength development rate between 28 d and 1 year was high.

**Figure 9:**
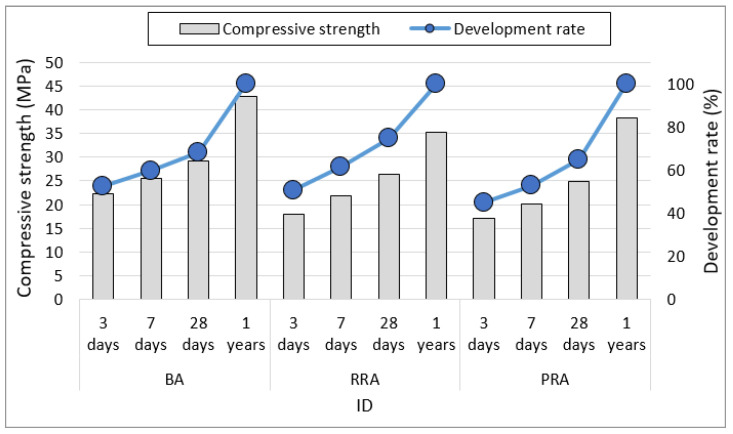
Compressive strength and development of concrete depending on aggregate.

Figure 9 illustrates the average compressive strength and strength development rate according to the aggregate type. BA attained the highest compressive strength, where BA FA0-W exhibited the highest one-year compressive strength of 60 MPa. PRA exhibited the lowest compressive strength until 28 d, but no significant difference was observed between the compressive strength of the PRA mix (38.9 MPa) and that of the BA mix (42.7 MPa) at 1 year when strength development was mostly determined to be completed. In the case of RRA, high strength development did not occur but was expected because of the stimulation of FA by the alkali components of the old paste. As the concrete strength was increased by cement paste development, further strength development was difficult because of the breakage of the old paste. For the utilization of the Jeju recycled aggregate, a certain level of quality improvement and clear setting of working strength are required.

### 3.4. Modulus of Elasticity

**Figure 10:**
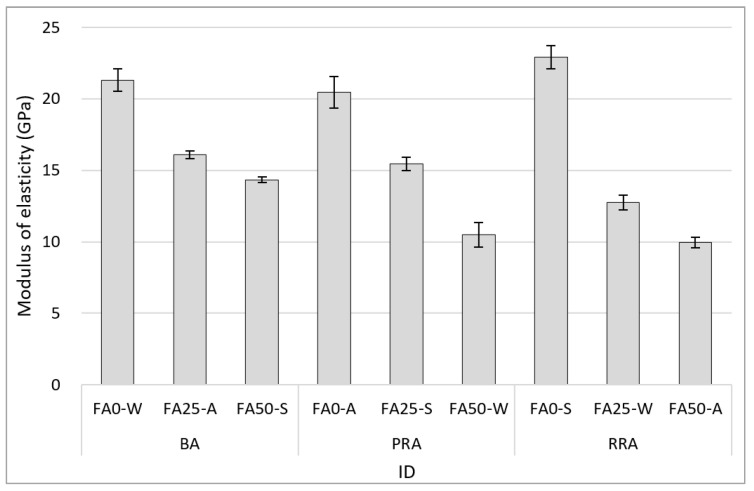
Modulus of elasticity of concrete.

Figure 10 illustrates the modulus of elasticity of hardened concrete containing B-RA at 28 d. The concrete variables were the aggregate type, FA substitution rate, and curing condition. The modulus of elasticity clearly decreased as the FA substitution rate increased. The modulus of elasticity apparently decreased in proportion to the reduction in compressive strength caused by the increase in FA substitution rate. Overall, the BA mix attained the highest modulus of elasticity, but PRA FA0-S exhibited the highest value of 22.9 GPa. This phenomenon likely occurred because the increase in filling performance resulting from using PRA with a high sphericity in the normal strength region affected the pore reduction, even though PRA FA0-S had a lower compressive strength than that of BA FA0-W. This tendency is consistent with the results of a previous domestic study on porous basalt recycled aggregate in Jeju Island; recycled aggregate concrete containing a certain level of old paste in the parent rock (basalt) with a relatively large number of pores (basalt) will not significantly affect the modulus of elasticity in the normal strength region [33].

**Figure 11:**
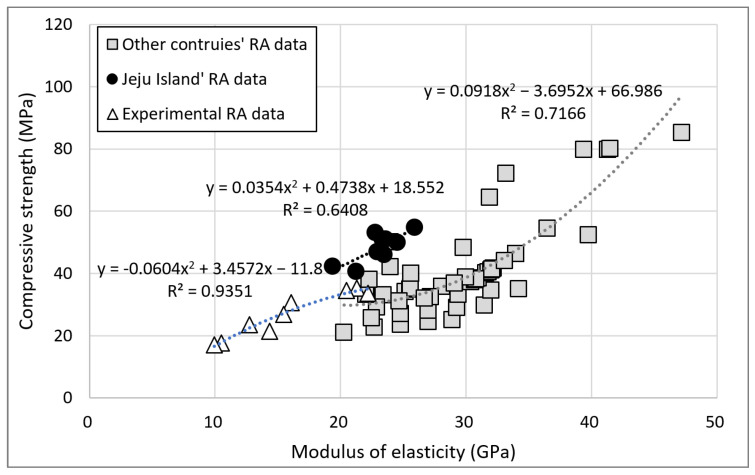
Relationship between compressive strength and the modulus of elasticity for domestic and overseas B-RA concrete.

Figure 11 illustrates the relationship between the modulus of elasticity and compressive strength for concrete with B-RA; the data were obtained from the literature [46,47,48,49,50,51,52,53,54,55,56,57,58,59,60,61,62,63]. The water absorption of most overseas basalt is less than 1%. Therefore, the recycled aggregate concrete comprising low-porosity basalt as the parent rock exhibited a high modulus of elasticity compared with compressive strength. For Jeju basalt, on the contrary, the modulus of elasticity was low compared with compressive strength because of high porosity with water absorption of approximately 3%. These results are similar to Jeju B-RA results; however, the slope of the trend line differed because of differences in the unit weight of cement.

### 3.5. Drying Shrinkage

**Figure 12:**
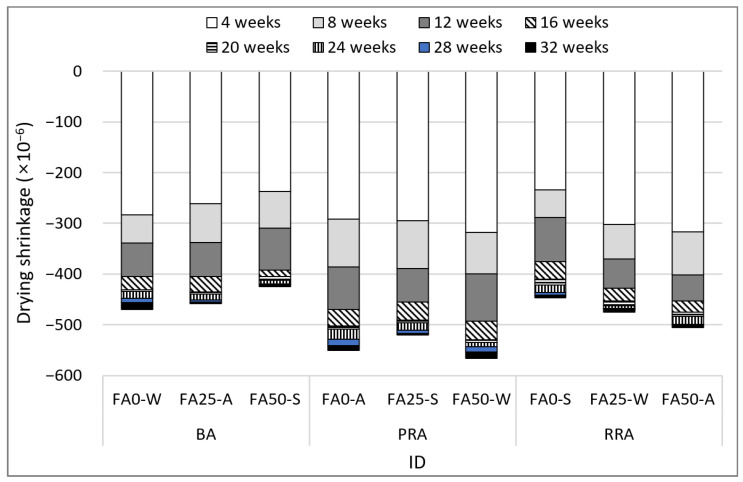
Drying shrinkage of concrete.

**Figure 13:**
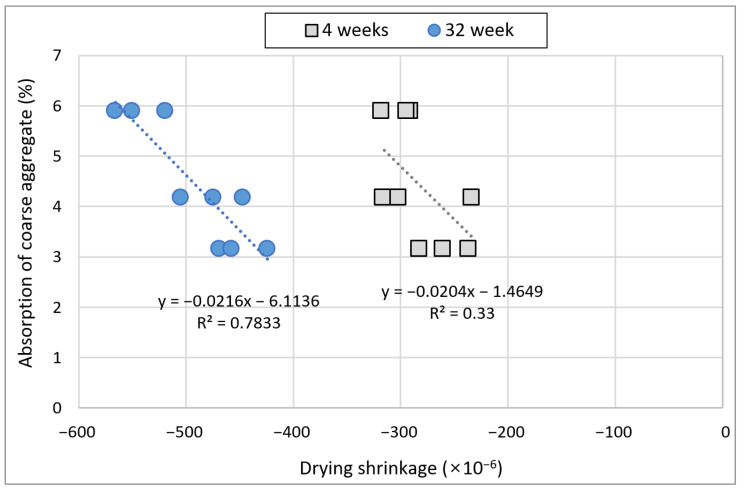
Relationship between drying shrinkage and absorption of coarse aggregate.

Figure 12 illustrates the drying shrinkage of concrete comprising B-RA. Shrinkage clearly decreased for the specimens subjected to steam curing. This phenomenon apparently occurred because a relatively large amount of moisture was used at the beginning as reaction was promoted at a high temperature of 60 °C during steam curing. In relation to the amount of FA used, drying shrinkage increased as the FA substitution rate increased for most specimens. This phenomenon likely occurred because the concrete was dried without the consumption of free water due to the slow reaction of FA. The use of recycled aggregate increased drying shrinkage, and no significant difference was found in drying shrinkage between the PRA and BA specimens. Figure 13 illustrates the relationship between drying shrinkage and the absorption of coarse aggregate. For the first four weeks with a large drying shrinkage, no clear drying shrinkage tendency was observed for the absorption of aggregate; likewise, no significant difference was observed in shrinkage depending on the absorption of recycled aggregate. Over time, the influence of the absorption of aggregate on drying shrinkage became clear. At 32 weeks, the difference in drying shrinkage between RRA (absorption: 5.9%) and PRA (absorption: 4.2%) increased as the tendency became clearer. Thus, the influence of the aggregate on drying shrinkage increased over time, and quality improvement considerably reduced drying shrinkage.

### 3.6. Freezing and Thawing and Pore Characteristics

**Figure 14:**
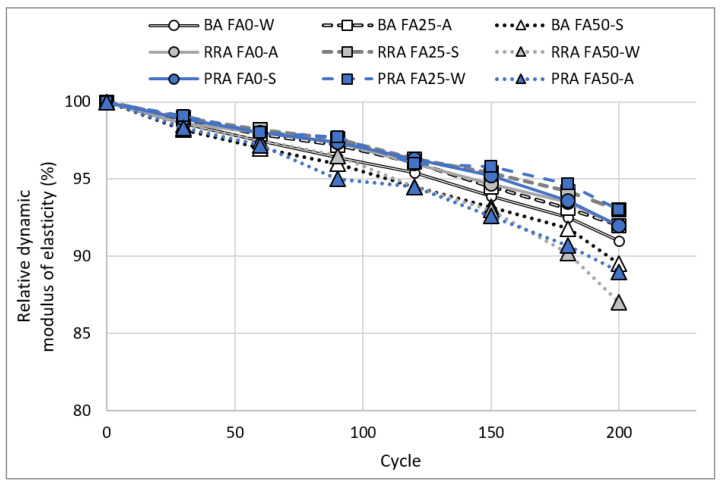
Relative dynamic modulus of elasticity of concrete depending rapid freezing and thawing.

Figure 14 illustrates the relative dynamic modulus of the elasticity of concrete comprising B-RA according to freezing and thawing. When the FA substitution rate was 25%, the relative dynamic modulus of elasticity increased until 200 freeze–thaw cycles were reached. This phenomenon occurred because the FA reacted with calcium hydroxide without extracting it from cement to the outside, and the matrix was reinforced by the pozzolanic reaction [64]. At an FA substitution rate of 50%, however, the relative dynamic modulus of elasticity increased regardless of the aggregate type. The increase occurred because freezing and thawing were affected by compressive strength, pore distribution, and micro-matrix. We estimate that the freeze–thaw resistance was reduced by low compressive strength at an FA substitution rate of 50%.

**Figure 15:**
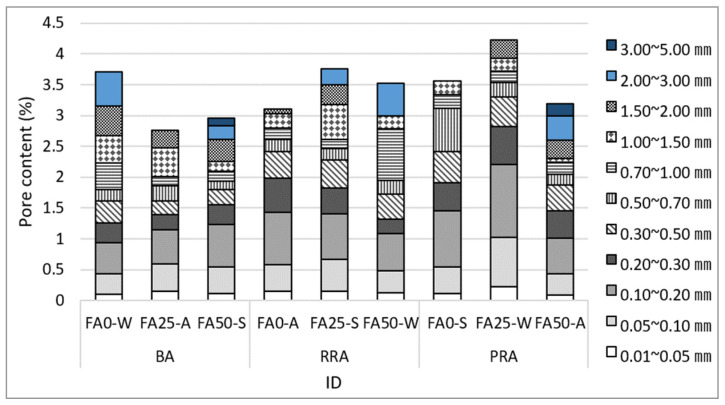
Pore distribution of hardened concrete.

**Figure 16:**
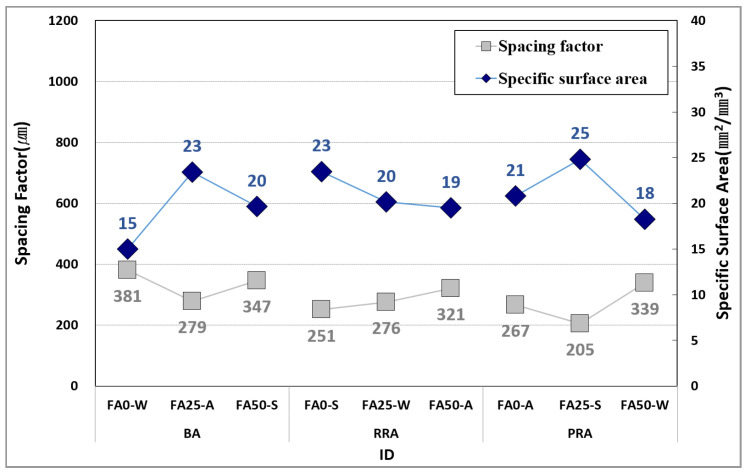
Spacing factor and specific surface area of concrete.

Figure 15 illustrates the pore distribution for the concrete specimens, and Figure 16 illustrates the spacing factor and specific surface area of concrete according to the concrete pore distribution. The pore content in concrete had the same tendency as the air content measured according to pores of 1 mm or less caused by entrained air. This phenomenon occurred because the entrained air in fresh concrete was hardened without being broken. Image analysis showed that the entrained air included large and small air bubbles in the basalt (including the one in the recycled aggregate) and old paste. Thus, the specimens that comprised RRA and PRA, which have higher absorption than that of BA, exhibited a relatively higher pore content.

**Figure 17:**
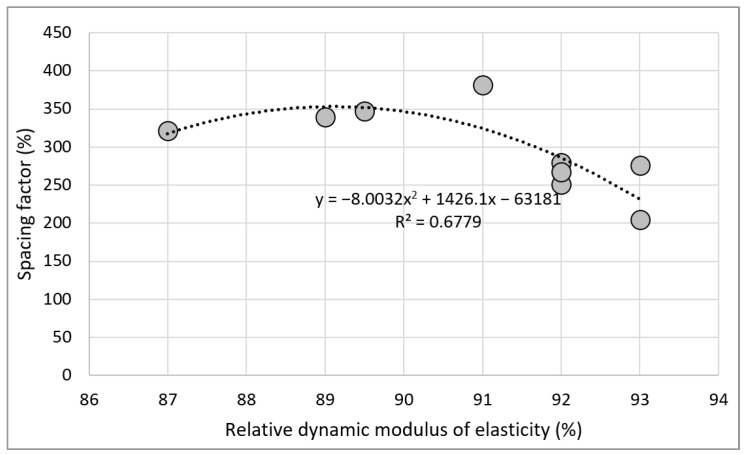
Spacing factor and relative dynamic modulus of elasticity of concrete.

According to the Mindess and Kansas DOT standards, the freeze–thaw resistance is high when the spacing factor is 250 µm or higher, and the specific surface area is 25 mm^2^/mm^3^ or larger [65]. The PRA FA25-W specimen satisfied these criteria and exhibited a high relative dynamic modulus of elasticity according to freezing and thawing. Figure 17 illustrates the relationship between the spacing factor and relative dynamic modulus of elasticity of concrete. Compared to the specific surface area, the spacing factor exhibited a high tendency with the relative dynamic modulus of elasticity. Although the specimen strength caused a deviation from the high tendency, the freeze–thaw resistance was high as the spacing factor decreased. In the case of recycled aggregate, the freeze–thaw resistance was high regardless of its quality. This phenomenon occurred because the pores included in the old paste of recycled aggregate reduced the spacing factor.

## 4. Conclusions

For the activation of porous basalt-based recycled aggregate (B-RA), the basic properties of concrete comprising B-RA were analyzed using three variables: quality of recycled aggregate, fly ash (FA) substitution rate, and water–cement ratio. The results of the study are as follows.

1. For the properties of fresh concrete, there was no decrease in the slump due to the influence of the recycled aggregate, but some air content increased due to the porous old paste of the recycled aggregate.

2. For concrete strength, the compressive strength did not show much difference depending on the quality of the recycled aggregate at 28 days age, but the compressive strength (60.6 MPa) of control specimen was the highest at 1 year age, while the strength decreased depending on the quality of the recycled aggregate. At the 1-year strength, the effect of curing conditions was significant, and the strength of recycled aggregate became 50 MPa due to improved quality.

3. The modulus of elasticity of concrete considerably decreased as the FA replacement rate increased at 28 days age. The use of improved recycled aggregates with high sphericity was attributed to the increase in the elastic modulus (22.9 GPa) by inducing the improvement of concrete fillability due to aggregates.

4. For the durability of concrete, steam curing tended to decrease drying shrinkage, which this is because blended water was consumed and a dense matrix was constructed due to high temperature heat and humidity in early curing. When the improved recycled aggregate was used, the drying shrinkage decreased over time at a level similar to that of basalt. The freeze–thaw resistance increased when the FA substitution rate was 25% and was improved by the use of recycled aggregate. This is because the use of fly ash drives the consumption of Ca(OH)_2_ and that of recycled aggregates improves the spacing factor.

5. Although the water absorption of the basalt-based recycled aggregate in the experiment did not reach the domestic standard of 3% or less for recycled aggregate, mechanical properties were equivalent to that of basalt combination when 50% was used by improving quality. If the basalt on the rough surface and the spherical recycled aggregate through quality improvement are mixed at less than 50%, it is believed that some durability can be improved by increasing the fillability of concrete and securing the spacing factor.

Although it is deemed necessary to further verify the properties of fresh concrete and various durability, the possibility of improving durability through the use of industrial by-product binder and steam curing is confirmed. Using this, it is judged that it can be used in concrete secondary products with general strength through management of curing and the use of industrial byproduct-based binders. It is also believed that this utilization will replace basalt, a natural resource of the region, and eco-friendly circulation with economic feasibility will be the basis for the system.

## Figures and Tables

**Table 1:** Experimental plan.

Aggregate Type	Replacement Ratio of Fly Ash (wt%)	Curing Condition
Basalt aggregate (BA)	0	Water curing (W)
Raw recycled aggregate and Basalt (RRA)	25	Air-dry curing (A)
Improved recycled aggregate and Basalt (PRA)	50	Steam and air-dry curing (S)

**Table 2:** Mix proportion of concrete.

ID	Air (%)	w/b (%)	s/a (%)	Unit Weight (kg/m^3^)							
				Water	Binder		Coarse Aggregate			Fine Aggregate	Total
					Cement	FA	Basalt	RRA	PRA		
BA FA0-W	5	43	40	163	380	-	1040	-	-	658	2241
BA FA25-A				163	285	95	1015	-	-	642	2201
BA FA50-S				163	190	190	990	-	-	626	2160
RRA FA0-A				163	380	-	520	440	-	658	2161
RRA FA25-S				163	285	95	508	429	-	642	2122
RRA FA50-W				163	190	190	495	418	-	626	2083
PRA FA0-S				163	380	-	520	-	485	658	2206
PRA FA25-W				163	285	95	508	-	473	642	2166
PRA FA50-A				163	190	190	495	-	461	626	2126

**Table 3:** Physical properties of cement.

Density (g/cm^3^)	Fineness (m^2^/kg)	Setting Time (min)		Compressive Strength (MPa)		
		Initial	Final	3 Days	7 Days	28 Days
3.15	382	45	375	23.0	29.3	42.5

**Table 4:** Physical properties of fly ash.t.

Density (g/cm^3^)	Fineness (m^2^/kg)	Flow Value Ratio (%)	Activity Index (%)	
			28 Days	91 Days
2.06	347	106	90	96

**Table 5:** Chemical composition of cement.

Type	CaO (%)	SiO_2_ (%)	Al_2_O_3_ (%)	Fe_2_O_3_ (%)	MgO (%)	Ig. Loss (%)	Other (%)	Total (%)
Cement	62.4	21.1	4.4	3.2	3.1	3.4	2.4	100
Fly ash	5.8	52.7	21.7	7.8	2.0	2.4	7.6	100

**Table 6:** Physical properties of coarse aggregates.

Aggregate Type	G_max_ (mm)	Oven-Dry Density (g/cm^3^)	Absorption (%)	Fineness Modulus	Bulk Density (kg/cm^3^)	Solid Content (%)
BA	25	2.60	3.16	6.90	1551.9	59.6
RRA	20	2.20	8.63	6.45	1369.0	56.4
PRA	20	2.43	5.19	6.45	1488.1	61.3

**Table 7:** Chemical composition of coarse aggregate.

Type	CaO (%)	SiO_2_ (%)	Al_2_O_3_ (%)	Fe_2_O_3_ (%)	MgO (%)	Na_2_O_3_ (%)	Other (%)	Total (%)
BA	9.01	48.36	13.95	11.88	9.02	2.92	4.86	100
RRA	31.4	36.2	9.86	7.84	5.97	1.77	6.99	100
PRA	16.5	45.3	12.13	10.08	7.66	2.41	5.95	100

## Data Availability

Not applicable.
